# Retraction: Evaluation of morphological traits of wheat varieties at germination stage under salinity stress

**DOI:** 10.1371/journal.pone.0273462

**Published:** 2022-08-31

**Authors:** The PLOS ONE Editors

The *PLOS ONE* Editors retract this article [1] because it was identified as one of a series of submissions for which we have concerns about authorship, competing interests, and peer review. We regret that the issues were not addressed prior to the article’s publication.

Additionally, after this article was published, it was noted that the first author’s name was spelled incorrectly. At the time of retraction, this article was republished to provide the correct name.

GM agreed with retraction. FG and SD did not agree with the retraction. MF and SHM responded but expressed neither agreement nor disagreement with the editorial decision.
